# The Linear Association of Chest Radiograph Opacification With Both Respiratory Physiology and Systemic Inflammation in Hospital In‐Patients With Covid‐19 Infection

**DOI:** 10.1111/crj.70056

**Published:** 2025-02-17

**Authors:** Colin J. Crooks, Dominick Shaw, Timothy R. Card, Iain Au‐Yong, Yutaro Higashi, Elisabetta Giannotti, Andrew W. Fogarty

**Affiliations:** ^1^ Nottingham Digestive Diseases Centre, School of Medicine University of Nottingham Nottingham UK; ^2^ NIHR Nottingham Biomedical Research Centre (BRC) Nottingham University Hospitals NHS Trust and the University of Nottingham Nottingham UK; ^3^ Nottingham University Hospitals NHS Trust Nottingham UK; ^4^ Division of Respiratory Medicine, School of Medicine University of Nottingham Nottingham UK; ^5^ Population and Lifespan Sciences, School of Medicine University of Nottingham Nottingham UK; ^6^ Department of Radiology Nottingham University Hospitals NHS Trust Nottingham UK

**Keywords:** chest radiograph, Covid‐19, Inflammation, physiology

## Abstract

**Background:**

Chest radiographs are generally used for diagnostic purposes. They also have potential to quantify disease severity. This analysis tested the hypothesis that there was an association between chest radiograph opacification and measures of respiratory physiological status and systemic inflammation in patients with Covid‐19 infection.

**Methods:**

Data on chest radiograph opacification were compared with concurrent measures of oxygen requirements and saturation and serum C‐reactive protein.

**Results:**

Data were available from 628 individuals. The median opacification on chest radiographs was 20% (interquartile range 5–45). This was associated both SFR (oxygen saturation/supplementary oxygen) with an *r* value of −0.38 (95% confidence intervals CI: −0.45 to −31, Pearson's correlation coefficient) and CRP (+0.33; 95% CI: +0.24 to +0.41, Pearson's correlation coefficient).

**Conclusion:**

Chest radiograph opacification scores are associated with both respiratory physiology status and systemic inflammation levels in patients with Covid‐19 infection.

## Graphical abstract

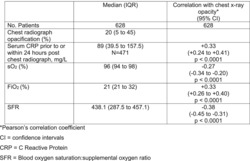

## 1 Introduction

The development of chest radiographs in the early 20th century was a landmark achievement that has been seamlessly integrated into mainstream clinical practice. Clinicians use their experience to integrate the information provided by the chest radiograph into the heuristic diagnostic process that utilises all available clinical information on the patient. This will inevitably include a degree of assessment of disease severity using a variety of parameters including the amount of chest radiograph opacification. A number of studies have explored the concept of radiograph opacity scoring systems for respiratory diseases [1] including in patients with Covid‐19 infection [2, 3, 4, 5]. One study from Italy reported that radiograph opacification was negatively associated with blood oxygen saturation and positive associated with systemic inflammation in patients with Covid‐19 infection [6].

We used data on a cohort of patients admitted to hospital with Covid‐19 infection to quantify the association between objective measures of chest radiograph opacification with respiratory failure severity and systemic inflammation.

## 2 Methods

Data were available for all adult patients admitted to Nottingham University Hospitals NHS Trust with a diagnosis of Covid‐19 infection from June 2020 to December 2021 [7]. Chest radiographs taken on admission to hospital from a subset of this cohort were scored by the reporting radiologist using a simple visual estimate of the total percentage of lung parenchymal opacification as previously described [8]. Data were extracted on the closest pulse oximeter oxygen saturation, supplementary oxygen requirements and serum C‐reactive protein (CRP) prior to the chest radiograph. If serum CRP was not measured prior to the chest radiograph, the first measurement within 24 h following the chest radiograph was used.

Pearson's product–moment correlation coefficients were calculated between the measures of chest radiograph opacification and both of the two measures of respiratory failure and systemic inflammation. Finally, we fitted separate probit models predicting percentage opacification for SFR and CRP, with adjustment for age and sex.

## 3 Results

Data were available from 628 individuals with observations available (Table 1). The median opacification score on chest radiographs was 20% (interquartile range 5–45). This was associated with both the measures of severity of respiratory failure and the measures of systemic inflammation. Specifically, the SFR (oxygen saturation/supplementary oxygen and the measure of severity of respiratory failure) was associated with the chest radiograph opacification score with an *r* value of −0.38 (95% confidence intervals CI: −0.45 to −31, *p* < 0.0001, Pearson's correlation coefficient), and the serum CRP (the measure of systemic inflammation) had a *r* value of +0.33 (95% CI: +0.24 to +0.41, *p* < 0.0001, Pearson's correlation coefficient). After adjusting for age and sex in multivariate probit models, both SFR and CRP were strongly associated with the chest X‐ray appearances (SFR, *p* < 0.0001 and CRP, *p* < 0.0001).

**TABLE 1:** The associations between chest X‐ray opacification and measures of respiratory failure severity and systemic inflammation.

	Median (IQR)	Correlation with chest X‐ray opacity^a^ (95% CI)
No. of patients	628	628
Chest radiograph opacification (%)	20 (5–45)	
Serum CRP prior to or within 24‐h post–chest radiograph, mg/L	89 (39.5–157.5) *N* = 471	+0.33 (+0.24 to +0.41) *p* < 0.0001
sO_2_ (%)	96 (94–98)	−0.27 (−0.34 to −0.20) *p* < 0.0001
FiO_2_ (%)	21 (21–32)	+0.33 (+0.26 to +0.40) *p* < 0.0001
SFR	438.1 (287.5–457.1)	−0.38 (−0.45 to −0.31) *p* < 0.0001

Abbreviations: CI, confidence intervals; CRP, C‐reactive protein; SFR, blood oxygen saturation:supplemental oxygen ratio.

^a^ Pearson's correlation coefficient.

## 4 Discussion

Our analysis quantified the nature and size of the associations between chest radiograph opacification and measures of both respiratory failure and systemic inflammation in patients with Covid‐19 infection.

The strength of this analysis is that data were collected on an unselected population of all patients admitted with Covid‐19 infection, with no awareness of the hypothesis to be tested, thus reducing the risk of bias. The quantification of the chest radiograph opacification by radiologists, again with no awareness of the hypothesis being tested, minimises the risk of bias. The analysis was done in patients with Covid‐19 infection and hence may not be generalisable to individuals outside this patient group. One limitation of the analysis was the absence of baseline premorbid radiographs to quantify preexisting opacification that is not due to Covid‐19 infection. This introduces the possibility of some measurement error for the radiograph opacification attributable to Covid‐19 infection, which may reduce the strength of some of the correlations we observed.

The analysis shows relatively strong associations between the extent of chest radiograph opacification and both the degree of respiratory failure and systemic inflammation. A correlation coefficient of 0.3 suggests that the opacification is associated with approximately 9% of the variance in respiratory failure and systemic inflammation observed in patients with Covid‐19 infection. This is of a similar magnitude to that reported in a population of patients with Covid‐19 infection from Italy [6]. The use of the SFR [9] provided a measure of hypoxaemia that has been validated in Covid‐19 patients [10] and is a prognostic measure of the risk of clinical deterioration [11]. Systemic serum CRP is also an independent risk factor for mortality in Covid‐19 patients [12].

## 5 Conclusion

These data demonstrate that as well as providing qualitative or diagnostic clinical information, chest radiographs' utility in patients with Covid‐19 infection extends to providing quantitative insights into concurrent disease severity, as measured in terms of both respiratory failure and systemic inflammation levels. This scoring complements and probably contributes to the ability of the chest radiograph scoring to contribute to the understanding of the clinical status of patients with Covid‐19 infection [8].

## Author Contributions

The concept was developed by AF and DS. I A‐Y, EG and YH provided scores for the radiographs. CC did the analysis. All authors contributed to the manuscript.

## Ethics Statement

Approval for this work was granted via an NUH Clinical Effectiveness Team audit (Reference: 21‐294C) and IRAS (REC: 20/WM/0142, project ID: 282490, Amendment No. SA02 20/07/21).

## Conflicts of Interest

The authors declare no conflicts of interest.

## Data Availability

These data are not available due to UK data governance legislation.
